# Highly selective fluorescent chemosensor for Zn^2+ ^derived from inorganic-organic hybrid magnetic core/shell Fe_3_O_4_@SiO_2 _nanoparticles

**DOI:** 10.1186/1556-276X-7-86

**Published:** 2012-01-25

**Authors:** Yujiao Wang, Xiaohong Peng, Jinmin Shi, Xiaoliang Tang, Jie Jiang, Weisheng Liu

**Affiliations:** 1 Key Laboratory of Nonferrous Metals Chemistry and Resources Utilization of Gansu Province and State Key Laboratory of Applied Organic Chemistry, College of Chemistry and Chemical Engineering, Lanzhou University, Lanzhou, 730000, People's Republic of China

## Abstract

Magnetic nanoparticles with attractive optical properties have been proposed for applications in such areas as separation and magnetic resonance imaging. In this paper, a simple and novel fluorescent sensor of Zn^2+ ^was designed with 3,5-di-tert-butyl-2-hydroxybenzaldehyde [DTH] covalently grafted onto the surface of magnetic core/shell Fe_3_O_4_@SiO_2 _nanoparticles [NPs] (DTH-Fe_3_O_4_@SiO_2 _NPs) using the silanol hydrolysis approach. The DTH-Fe_3_O_4_@SiO_2 _inorganic-organic hybrid material was characterized by transmission electron microscopy, dynamic light scattering, X-ray power diffraction, diffuse reflectance infrared Fourier transform, UV-visible absorption and emission spectrometry. The compound DTH exhibited fluorescence response towards Zn^2+ ^and Mg^2+ ^ions, but the DTH-Fe_3_O_4_@SiO_2 _NPs only effectively recognized Zn^2+ ^ion by significant fluorescent enhancement in the presence of various ions, which is due to the restriction of the N-C rotation of DTH-Fe_3_O_4_@SiO_2 _NPs and the formation of the rigid plane with conjugation when the DTH-Fe_3_O_4_@SiO_2 _is coordinated with Zn^2+^. Moreover, this DTH-Fe_3_O_4_@SiO_2 _fluorescent chemosensor also displayed superparamagnetic properties, and thus, it can be recycled by magnetic attraction.

## Background

Zinc is the second abundant transition metal ion in the human body, which plays a vital role in various biological processes, such as gene expression [1], apoptosis [2], enzyme regulation [3], and neurotransmission [4,5]. It is also believed that the Zn^2+ ^homeostasis may have some bearing on the pathology of Alzheimer's disease and other neurological problems [6-8]. Therefore, there is an urgency to develop approaches to detect Zn^2+ ^*in vivo*. Besides, techniques for the separation and removal of metal ions and additives in the detection process are very important to prevent poisoning in environmental and biological fields. Conventional analytical methods including atomic absorption spectrophotometry [9], inductively coupled plasma atomic emission spectrometry [10], and electrochemical method [11] can hardly be applied for Zn^2+ ^ion detection in biological systems due to their complicated pretreatment steps and expensive equipment. Hence, for convenience in future *in vivo *applications, various fluorescent probes based on small molecules have been designed. They were fairly efficient as reported [12-22]; however, the small molecules would be toxic [23], and it is impossible to recover or remove them from organisms [24]. The limitation of recoverability blocked the practical applications of small molecular fluorescent probes. To resolve this challenge, the inorganic supports incorporated with small molecular fluorescent probes were applied for the improvement on recoverability.

Various mesoscopic or nanoscopic materials can be acted as the inorganic supports in the design of fluorescent probes, including magnetic nanoparticles, nanotubes, mesoporous silica, metal nanoparticles, and TiO_2 _[25-34]. Among all these inorganic materials, magnetic silica core/shell nanoparticles have advantages over other competitors for biological and environmental applications [35-41]. Firstly, they could be simply separated or recovered via external magnetic field. Besides, with magnetic silica core/shell nanoparticles as delivery, their low toxicity and biocompatibility also had advantages for the design of biological fluorescent probes. Furthermore, the silica shell around magnetic core has large surface area, and it can be grafted by fluorescent probes. Therefore, to develop nontoxic, biocompatible, and recoverable fluorimetric Zn^2+ ^sensors, introducing the magnetic silica nanoparticles with small molecular fluorescent probes incorporated is very necessary and highly desirable.

**Figure 1:**
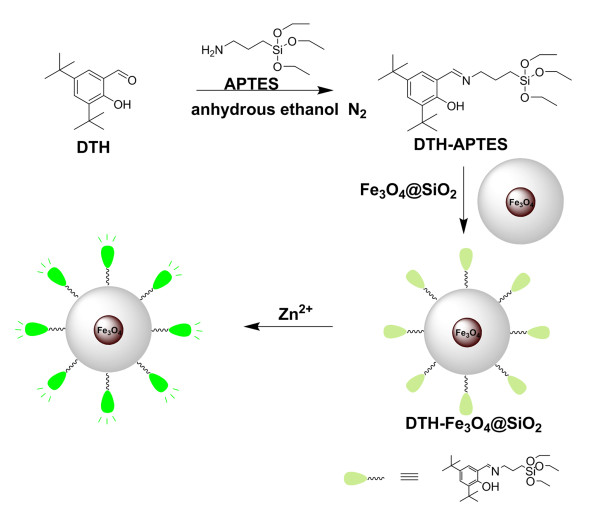
**Syntheses of DTH-APTES and DTH-Fe_3_O_4_@SiO_2_**.

In this work, we designed and synthesized a magnetic recoverable fluorescence Zn^2+ ^sensor based on 3,5-di-tert-butyl-2-hydroxybenzaldehyde [DTH] covalently grafted onto Fe_3_O_4_@SiO_2 _nanoparticles [NPs] (DTH-Fe_3_O_4_@SiO_2_) to provide highly selective fluorescence changes and efficient magnetic recoverability (Figure 1). This Zn^2+^-selective fluorescent switch of the immobilized chemosensors displayed excellent reversibility, combined with its superparamagnetic property, enabling the recovery of material and repeated uses for Zn^2+ ^sensing.

## Experimental details

### Materials and methods

All reagents are purchased commercially. Besides, ethanol was used after purification by standard methods. Other chemicals were used as received without further purification.

Thermal gravimetric analysis [TGA] (P.E. Diamond TG/DTA/SPAECTRUN ONE thermal analyzer, PerkinElmer Inc., Waltham, MA, USA), dynamic light scattering (BI-200SM, Brookhaven Instruments Corporation, Holtsville, NY, USA), transmission electron microscopy [TEM] (Tecnai G^2 ^F30, 300 kV, FEI Company, OR, USA), and energy-dispersive X-ray spectrometer [EDX] were used to characterize the materials. X-ray diffraction [XRD] pattern of the synthesized products was recorded with a Rigaku D/MAX 2400 X-ray diffractometer (Tokyo, Japan) using Cu *Kα *radiation (*λ *= 0.154056 Å). The scan range (2*θ*) was from 10° to 80°. Solid-state infrared [IR] using diffuse-reflectance infrared Fourier transform [DRIFT] spectroscopy was performed in the 400- to 4,000-cm^-1 ^region using a Bruker Vertex 70v (Bremen, Germany) and IR-grade KBr (Sigma-Aldrich Corporation, St. Louis, MO, USA) as the internal standard. ^1^H NMR and ^13^C NMR spectra were measured on a Bruker DRX 400 spectrometer in a CDCl_3 _solution with TMS as the internal standard. Chemical shift multiplicities are reported as s = singlet, t = triplet, q = quartet, and m = multiplet. Mass spectra were recorded on a Bruker Daltonics esquire6000 mass spectrometer. UV absorption spectra were recorded on a Varian Cary 100 spectrophotometer (Palo Alto, CA, USA) using quartz cells of 1.0-cm path length. Fluorescence measurements were made on a Hitachi F-4500 spectrophotometer (Tokyo, Japan) and a Shimadzu RF-540 spectrofluorophotometer (Chorley, UK) equipped with quartz cuvettes of 1.0-cm path length with a xenon lamp as the excitation source. An excitation and emission slit of 10.0 nm was used for the measurements in the solution state. All spectrophotometric titrations were performed with a suspension of the sample dispersed in ethanol.

#### Synthesis of Fe_3_O_4_@SiO_2 _NPs

Fe_3_O_4_@SiO_2 _NPs were synthesized according to the study of Nigam et al. [42]. The process can be briefly described in the following two steps: (1) FeCl_2 _and FeCl_3 _(molar ratio, 1:2) were added to a concentrated solution of base (25% ammonium hydroxide) under N_2_. The solution was mechanically stirred for 1 h at 20°C and then heated at 70°C for 1 h. The mixture was then stirred for 30 min at 90°C upon addition of citric acid (0.5 g/ml). After cooling the reaction mixture to room temperature, the magnetite NPs were obtained by permanent magnet, and then it was rinsed with deionized water to remove excess citric acid and other nonmagnetic particles thoroughly. (2) Then, the magnetite NPs were further coated with a thin silica layer via a modified Stöber method [43] to obtain stable Fe_3_O_4_@SiO_2_. Tetraethyl orthosilicate was hydrolyzed with magnetic NPs as seeds in an ethanol/water mixture. The resulting silica-coated magnetite NPs with an average diameter of 60 to 70 nm were used.

#### Synthesis of DTH-Fe_3_O_4_@SiO_2 _NPs

As shown in Figure 1, the synthetic procedure for 2,4-di-tert-butyl-6-((3-(triethoxysilyl)propylimino)methyl)phenol [DTH-APTES] followed the method previously described in the literatures [44,45]. DTH (234 mg, 1 mmol) and (3-aminopropyl) triethoxysilane [APTES] (221 mg, 1 mmol) were mixed in dry ethanol (15 mL) at room temperature. Then, the solution was refluxed for 3 h under N_2_. After that, the solvent was evaporated, and the crude product was further purified by flash column chromatography (silica gel, ethyl acetate/petroleum ether 1:2) to produce 371 mg (84.9%) of DTH-APTES as yellow oil. ESI-MS: *m/z *438.5 (M + H^+^). ^1^H NMR: (400 MHz, CDCl_3_): δ (ppm) 0.69 (t, 2H, CH_2_Si); 1.22 (t, 9H, CH_3_); 1.30 (s, 9H, C(CH_3_)_3_); 1.43 (s, 9H, C(CH_3_)_3_); 1.82 (m, 2H, CH_2_); 3.58 (t, 2H, NCH_2_); 3.82 (q, 6H, SiOCH_2_); 7.07, 7.36 (d, 2H, Ar); 8.34 (s, 1H, HC = N). ^13^C NMR (100 MHz, CDCl_3_): 7.92 (CH_2_Si); 18.30 (CH_3_); 24.38, 29.40, 29.70, 31.50 (CH_3_); 34.11 (C), 35.01 (C); 58.41 (CH_2_); 62.08 (CH_2_); 117.83, 125.69, 126.66, 136.65, 139.75, 158.27 (Ar); 165.80 (C = N). FT-IR (KBr pellet) (cm^-1^): 1,637 (*ν*_C = N_), 1,275-1,252 (*ν*_C-O_), 1,596-1,342 (*ν*_C = C_), 1,106-1,085 (*ν*_Si-O_).

One hundred milligrams of dried Fe_3_O_4_@SiO_2 _NPs and 356 mg (0.81 mmol) of DTH-APTES were suspended in 10 mL of anhydrous ethanol. The mixture was refluxed for 8 h at 80°C under N_2 _to obtain DTH-Fe_3_O_4_@SiO_2_. The nanoparticles were collected by centrifugation and repeatedly washed with anhydrous ethanol thoroughly. Unreacted organic molecules were removed completely and monitored by the fluorescence of the upper liquid. Then, the DTH-Fe_3_O_4_@SiO_2 _NPs were finally dried under vacuum over night. About 2.81% DTH-APTES in the precursors was finally grafted on the NPs, and the rest could be recycled if no hydrolysis occurred.

## Results and discussion

### Characterization of DTH-Fe_3_O_4_@SiO_2_

**Figure 2:**
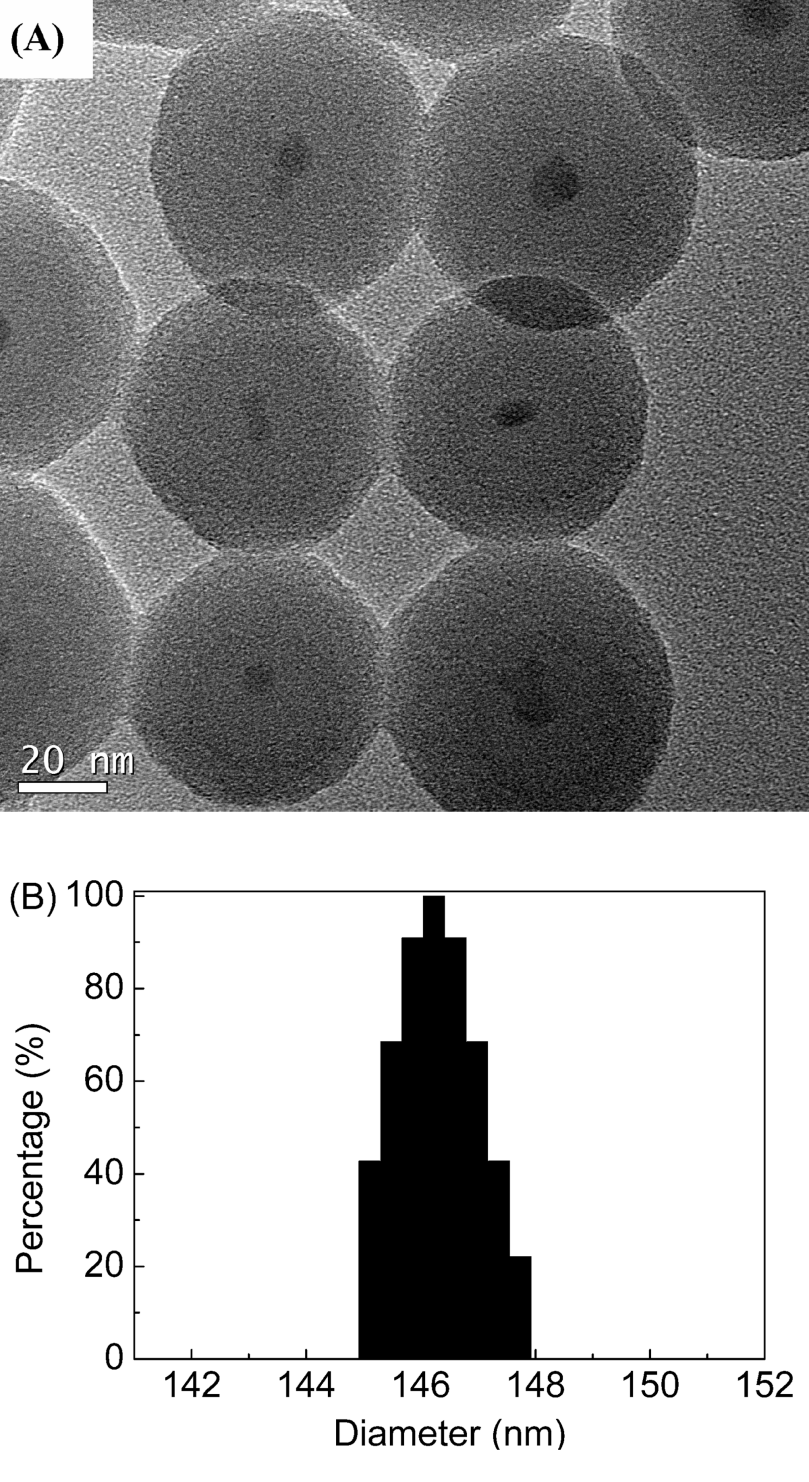
**TEM image (A) and the particle size histogram from DLS (B) of DTH-Fe_3_O_4_@SiO_2_**.

The TEM image (Figure 2A) of DTH-Fe_3_O_4_@SiO_2 _reveals that iron oxide NPs have entrapped in the silica shell successfully, in which the core/shell structures are in a narrow size distribution of 60 to 70 nm [46,47], and the diameter of the magnetic core is about 10 nm. The weight ratio of iron vs. silicon was measured to be 2.63:38.94 by EDX. Hence, according to TGA, each magnetic NP has about 6,000 DTH-APTES molecules grafted (see Additional file 1). More importantly, the right size of magnetic core/shell NPs smaller than 100 nm is an advantage for their good dispersibility. In addition, an inert silica coating on the surface of magnetite nanoparticles prevents their aggregation in liquid [48]. Hence, such a good performance on the dispersibility can improve their chemical stability and provide better protection against toxicity.

In addition, dynamic light scattering [DLS] was performed to further reveal the colloidal stability of NPs. According to DLS results (Figure 2B), DTH-Fe_3_O_4_@SiO_2 _presents good stabilization and a narrow size distribution with peak centered at 147 nm, confirming its good stabilization in ethanol. In a common sense, the diameter achieved by DLS is mostly higher than the one observed in TEM since the size of NPs identified by DLS includes the grafted molecules' steric hindering and the hydrodynamic radius of first few solvent layers [49-51]. Besides, according to the calculated size of DTH-APTES which covalently grafted on the surface of Fe_3_O_4_@SiO_2_, the grafted molecules' steric hindering could increase the diameter by about 2.72 nm.

**Figure 3:**
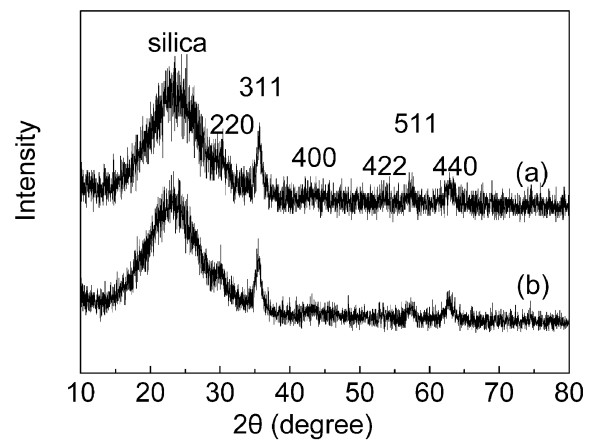
**XRD patterns of Fe_3_O_4_@SiO_2 _(a) and DTH-Fe_3_O_4_@SiO_2 _(b)**.

Figure 3 shows the XRD powder diffraction patterns of two NPs for the identification of Fe_3_O_4 _in core/shell NPs. XRD patterns of the synthesized Fe_3_O_4_@SiO_2 _(a) and DTH-Fe_3_O_4_@SiO_2 _(b) display relative diffraction peaks in the 2*θ *region of 10° to 80°. We could find that XRD patterns show very low intensities for the peaks attributed to the Fe_3_O_4 _cores, due to the coating of amorphous silica shell, which deduced the efficient content of Fe_3_O_4 _cores and then affected the peak intensities. However, the diffraction peaks of DTH-Fe_3_O_4_@SiO_2 _still maintain the same position as the magnetite core (Figure S1 in Additional file 1) [52]. The six characteristic diffraction peaks in Figure 3 can be indexed to (220), (311), (400), (422), (511), and (440), which well agree with the database of magnetite in the Joint Committee on Powder Diffraction Standards [JCPDS] (JCPDS card: 19-629) file [42,46,53,54]. Also, the broad XRD peak at a low diffraction angle of 20° to 30° corresponds to the amorphous-state SiO_2 _shells surrounding the Fe_3_O_4 _NPs [53].

**Figure 4:**
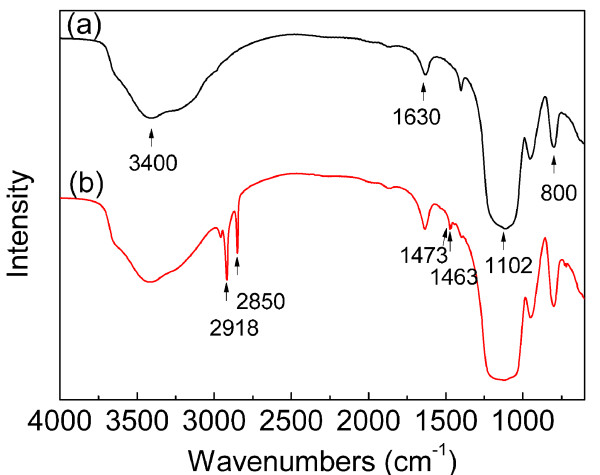
**DRIFT spectra of Fe_3_O_4_@SiO_2 _(a) and DTH-Fe_3_O_4_@SiO_2 _(b)**.

The successful conjugation of DTH onto the surface of the Fe_3_O_4_@SiO_2 _NPs can be confirmed by DRIFT (Figure 4). The bands at 3,400 to 3,500 cm^-1 ^and 1,000 to 1,250 cm^-1 ^are due to -OH stretching on silanol [55]. It indicates that not all the silanol on Fe_3_O_4_@SiO_2 _NPs have been covalently modified. The band at 1,630 cm^-1 ^represents the bending mode of -OH vibrations [56]. DTH-Fe_3_O_4_@SiO_2 _(see Figure 1) has additional peaks at 2,918 and 2,850 cm^-1 ^that correspond to the -CH vibration of aliphatic and aromatic groups [28,57,58]. The bands at 1,473 and 1,463 cm^-1 ^of DTH-Fe_3_O_4_@SiO_2 _are probably due to the bending vibrations of -CH_3_, which come from the DTH part [59]. According to the spectra of Fe_3_O_4_@SiO_2 _and DTH-Fe_3_O_4_@SiO_2_, the bands which appear as broad and strong and are centered at 1,102 (*ν*_as_) and 800 cm^-1 ^can be attributed to the siloxane (-Si-O-Si-) [60]. These results support the presence of the organic DTH-APTES in the magnetic material DTH-Fe_3_O_4_@SiO_2_.

**Figure 5:**
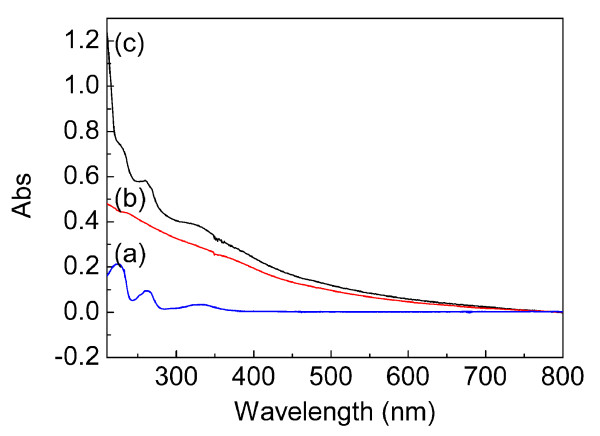
**UV-Vis spectra of DTH-APTES (a), Fe_3_O_4_@SiO_2 _(b), and DTH-Fe_3_O_4_@SiO_2 _(c)**.

The UV-visible [UV-Vis] spectra of DTH-APTES (1.0 × 10^-5 ^M), Fe_3_O_4_@SiO_2 _(0.3 g/L), and DTH-Fe_3_O_4_@SiO_2 _(0.3 g/L) can provide further evidence on the grafting of DTH onto the surface of the Fe_3_O_4_@SiO_2 _NPs (Figure 5). Compared to Fe_3_O_4_@SiO_2 _(b), a new absorption band centered at about 330 nm of DTH-Fe_3_O_4_@SiO_2 _can be attributed to the typical electronic transition of an aromatic ring and -C = N- conjugate system in a Schiff base molecule [29]. This result can also imply the successful immobilization of DTH-APTES onto the magnetic core/shell NPs.

**Figure 6:**
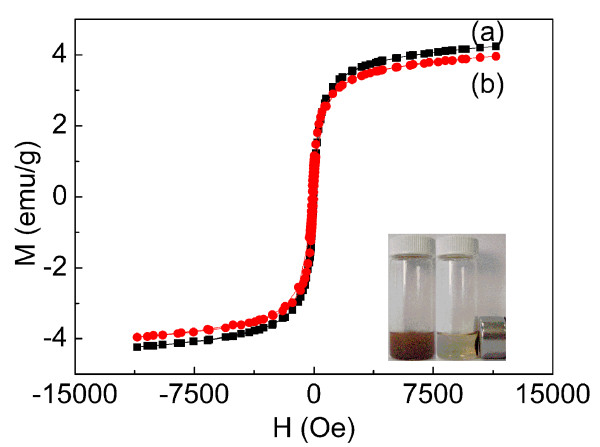
**Magnetization curves of the Fe_3_O_4_@SiO_2 _(a) and DTH-Fe_3_O_4_@SiO_2 _(b)**. Inset shows that DTH-Fe_3_O_4_@SiO_2 _was dispersed to an external magnet in ethanol.

The superparamagnetic property of the magnetic NPs plays a vital role for its biological application. Figure 6 shows the magnetization curves of the Fe_3_O_4_@SiO_2 _and DTH-Fe_3_O_4_@SiO_2 _which were investigated with a vibrating sample magnetometer tuned from -15,000 to 15,000 Oe at 300 K. The result was consistent with the conclusion that magnetic Fe_3_O_4 _NPs smaller than 30 nm are usually superparamagnetic at room temperature [47]. The saturation magnetization value for synthesized DTH-Fe_3_O_4_@SiO_2 _is about 3.96 emu/g. The saturation magnetization value for Fe_3_O_4_@SiO_2 _support was measured to be 4.24 emu/g. Considering the grafting rate of 7.64% (according to TGA, Figure S2 and Table S1 in Additional file 1), the difference of saturation magnetization values between DTH-Fe_3_O_4_@SiO_2 _and its support could be due to the decreased weight ratio of magnetic support after grafting. More importantly, from the hysteresis loops of Fe_3_O_4_@SiO_2 _NPs and the DTH-Fe_3_O_4_@SiO_2 _NPs, it can be found that both exhibited superparamagnetic properties for no remanence was observed when the applied magnetic field was removed. These phenomena were due to the fact that the magnetite core is smaller than 30 nm in core/shell NPs (Figure 2A). As a result of this superparamagnetic property, DTH-Fe_3_O_4_@SiO_2 _had a reversal magnetic responsivity. It could be easily separated from dispersion after only 5 min using a magnet (Figure 6, inset) and then redispersed by mild agitation when the magnet was removed. The reversal magnetic responsivity of DTH-Fe_3_O_4_@SiO_2 _would be a key factor when evaluating their recoverability [61]. The magnetic separation capability of DTH-Fe_3_O_4_@SiO_2 _NPs and the reversibility of the combination between DTH-Fe_3_O_4_@SiO_2 _and Zn^2+ ^could also provide a simple and efficient route to separate Zn^2+ ^rather than through filtration approach (see Figure 6 inset).

### Fluorescence response of DTH-Fe_3_O_4_@SiO_2_

**Figure 7:**
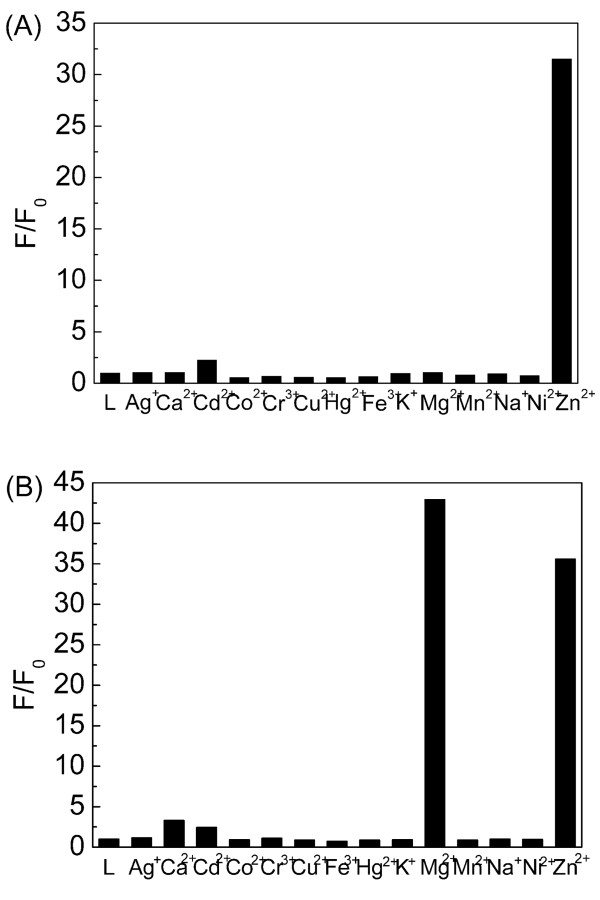
**Fluorescence response of DTH-Fe_3_O_4_@SiO_2 _(A) and DTH (B) to various cations**. Excitation wavelength was 397 nm. Spectra were recorded every 25 min after adding Zn^2+^.

To verify its fluorescence response towards various metal ions, we investigated fluorescence properties of DTH-Fe_3_O_4_@SiO_2 _NPs (0.3 g/L, containing 5.2 × 10^-5 ^M DTH-APTES according to TGA in Figure S2 and Table S1 in Additional file 1) towards various metal ions Ag^+^, Ca^2+^, Cd^2+^, Co^2+^, Cr^3+^, Cu^2+^, Fe^3+^, Hg^2+^, K^+^, Mg^2+^, Mn^2+^, Na^+^, Ni^2+^, and Zn^2+ ^in ethanol solution (all as perchlorates, 1.0 × 10^-4 ^M). As shown in Figure 7A, DTH-Fe_3_O_4_@SiO_2 _NPs exhibited significant 'off-on' changes in fluorescence emission only for Zn^2+^, but not for the others. It is noted that Cd^2+ ^with a *d*^10 ^electron configuration, which often exhibited coordination properties similar to Zn^2+ ^[19], do not influence the fluorescence intensity of DTH-Fe_3_O_4_@SiO_2 _NPs significantly. As a comparison, DTH (1.0 × 10^-5 ^M) exhibited fluorescence response towards both Zn^2+ ^and Mg^2+ ^ions (1.0 × 10^-4 ^M) in the same solution, which is not as selective as DTH-Fe_3_O_4_@SiO_2 _for Zn^2+ ^detection (Figure 7B). Compared to the single aldehyde DTH, the origin of selectivity for DTH-Fe_3_O_4_@SiO_2 _may come from its Schiff base structure, which prefers to coordinate with Zn^2+ ^under the interference of Mg^2+^.

The remarkable increase of fluorescence intensity can be explained as follows: DTH-Fe_3_O_4_@SiO_2 _is poorly fluorescent due to the rotation of the N-C bond of DTH-APTES part. When stably chelated with Zn^2+^, the N-C rotation of DTH-APTES part is restricted and the rigid plane with conjugation is formed and the fluorescence enhanced, which consists of our previous work [62]. The emission spectra of DTH-Fe_3_O_4_@SiO_2_, which is excited at 397 nm, exhibit the emission maximum at 452 nm with a low quantum yield (*Φ *= 0.0042) at room temperature in ethanol. Upon the addition of excess Zn^2+^, the fluorescence intensity of DTH-Fe_3_O_4_@SiO_2 _increased by more than 25-fold, the emission maximum shifts from 452 to 470 nm, and the quantum yield (*Φ *= 0.11) results in a 26-fold increase.

**Figure 8:**
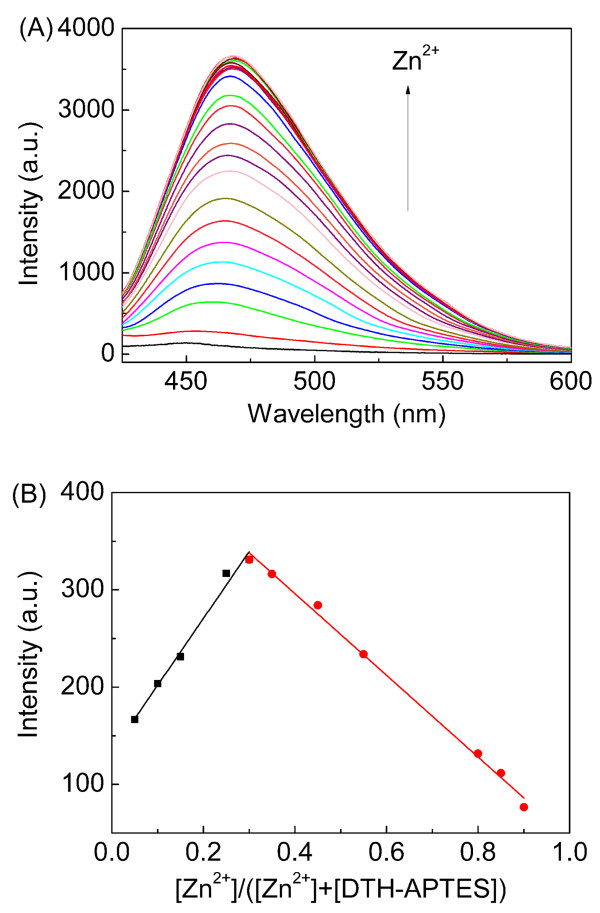
**Fluorescence titrations and Job's plot**. (**A**) Fluorescence titrations of DTH-Fe_3_O_4_@SiO_2 _with Zn^2+^. (**B**) Job's plot of DTH-APTES with Zn^2+^. Spectra were recorded every 25 min after adding Zn^2+^.

As illustrated in Figure 8A, the fluorescence emission of DTH-Fe_3_O_4_@SiO_2 _(0.3 g/L) increases gradually when adding various concentrations (0 to 30 μM) of Zn^2+ ^in ethanol, indicating that Zn^2+ ^is quantitatively bound to the Schiff base moiety attached to the NPs. Fluorescence titration experiment suggests that the association constant (*K_d_*) for Zn^2+ ^binding to DTH-Fe_3_O_4_@SiO_2 _is calculated to be 51.08 M^-2 ^(log *K *= 1.71; Figure 8A). Job's plot suggested a 1:2 binding ratio for Zn^2+ ^with DTH-APTES (Figure 8B).

**Figure 9:**
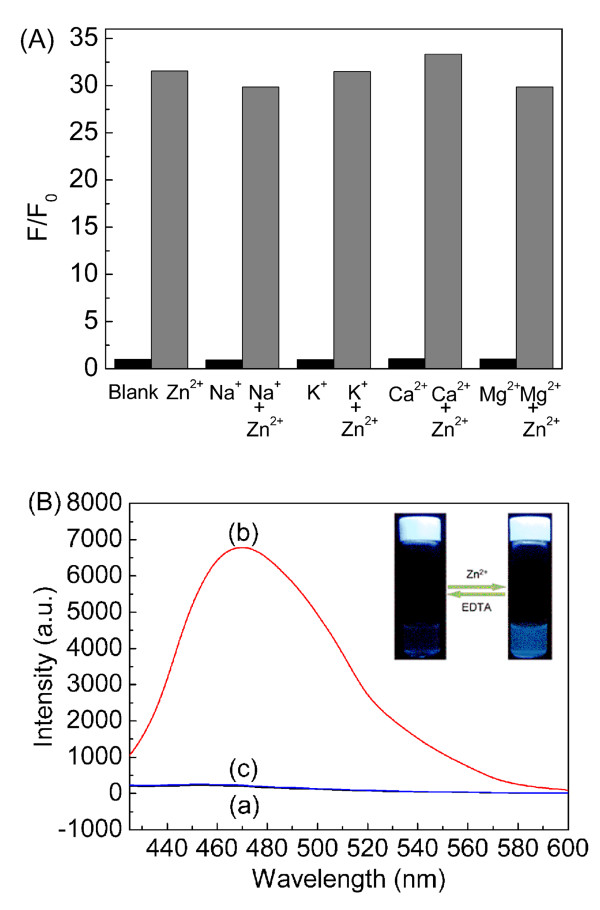
**Competition of DTH-Fe_3_O_4_@SiO_2 _towards cations and reversibility of DTH-Fe_3_O_4_@SiO_2 _towards Zn^2+^**. (**A**) Fluorescent emission changes of DTH-Fe_3_O_4_@SiO_2 _(0.3 g/L) upon addition of 1, blank; 2, Zn^2+^; 3, Na^+^; 4, Na^+ ^+ Zn^2+^; 5, K^+^; 6, K^+ ^+ Zn^2+^; 7, Ca^2+^; 8, Ca^2+ ^+ Zn^2+^; 9, Mg^2+^; and 10, Mg^2+ ^+ Zn^2+ ^(each metal ion is 100 μM) in ethanol at room temperature. (**B**) Fluorescence spectra of DTH-Fe_3_O_4_@SiO_2 _(0.3 g/L) in (a) without, (b) with Zn^2+ ^(1.0 × 10^-4 ^M), and (c) after treatment with EDTA (2.5 × 10^-4 ^M) in (b) solution. The inset picture shows the photograph of DTH-Fe_3_O_4_@SiO_2 _with Zn^2+ ^by treatment of EDTA (2.5 × 10^-4 ^M) under a 365-nm UV light.

The competition experiments indicated that the presence of most metal ions, especially Na^+^, K^+^, Ca^2+^, and Mg^2+^, which are abundant in the biological environment, had a negligible effect on Zn^2+ ^sensing (Figure 9A). Since Cr^3+^, Cu^2+^, Fe^3+^, and Hg^2+ ^also appeared to bind DTH-Fe_3_O_4_@SiO_2 _sensors (Figure S3 in Additional file 1), they quenched the fluorescence of the Zn^2+^-DTH-Fe_3_O_4_@SiO_2_, owing to an electron or energy transfer between the metal cation and fluorophore known as the fluorescence quenching mechanism [63-66]. The fluorescence enhancement that occurred upon exposure to Zn^2+ ^was fully reversible as the addition of EDTA (2.5 × 10^-4 ^M; Figure 9B and inset) restored the emission band. Combined with its magnetic property, the results above implied that DTH-Fe_3_O_4_@SiO_2 _was considerably applicable to some field as a new inorganic-organic hybrid sensor for the Zn^2+ ^ion.

**Figure 10:**
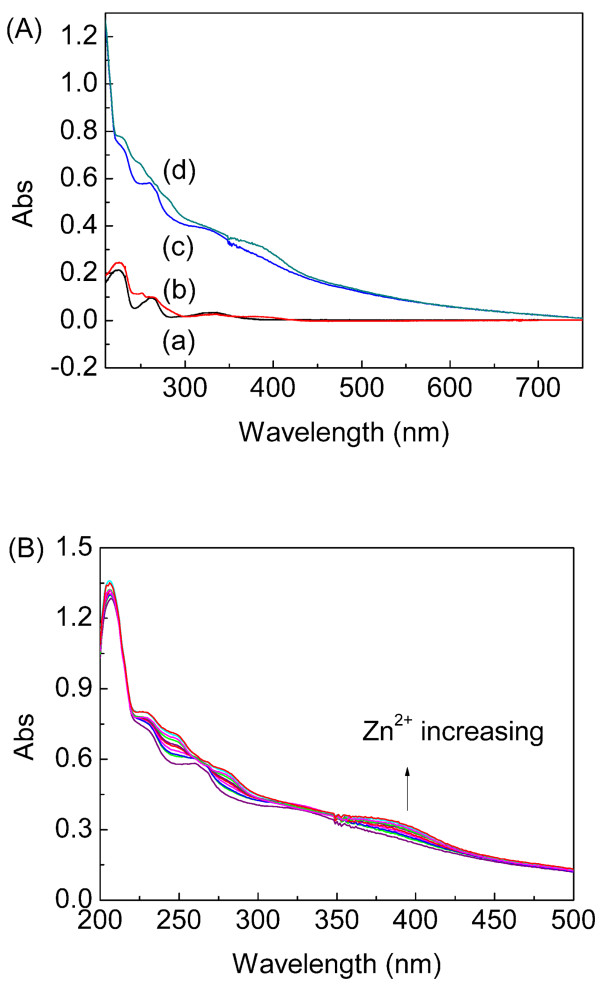
**UV-Vis spectra**. (**A**) Absorption spectra of (a) DTH-APTES (1.0 × 10^-5 ^M), (b) DTH-APTES + Zn^2+ ^(1.0 × 10^-4 ^M), (c) DTH-Fe_3_O_4_@SiO_2 _(0.3 g/L), and (d) DTH-Fe_3_O_4_@SiO_2 _(0.3 g/L) + Zn^2+ ^(1.0 × 10^-4 ^M) in ethanol. (**B**) UV-Vis spectra of DTH-Fe_3_O_4_@SiO_2 _(0.3 g/L) in ethanol in the presence of different amounts of Zn^2+ ^(0 to 240 μM).

Figure 10A depicts the UV-Vis spectra of DTH-APTES (10 μM), DTH-APTES (10 μM) + Zn^2+ ^(100 μM), DTH-Fe_3_O_4_@SiO_2 _(0.3 g/L), and DTH-Fe_3_O_4_@SiO_2 _(0.3 g/L) + Zn^2+ ^(100 μM). It can be seen that the absorbance peaks at around 390 nm are formed when Zn^2+ ^is added in both DTH-APTES and DTH-Fe_3_O_4_@SiO_2 _systems. The absorption spectra of DTH-Fe_3_O_4_@SiO_2 _(0.3 g/L) in the presence of various concentrations of Zn^2+ ^(0 to 240 μM) were investigated in ethanol at room temperature, as shown in Figure 10B. When Zn^2+ ^was added gradually, the absorbance of DTH-Fe_3_O_4_@SiO_2 _at 390 nm gradually increases, which indicated that DTH-Fe_3_O_4_@SiO_2 _NPs coordinated with Zn^2+ ^gradually.

## Conclusions

In summary, we have successfully designed and synthesized functionalized magnetic core/shell Fe_3_O_4_@SiO_2 _NPs (DTH-Fe_3_O_4_@SiO_2 _NPs) which could act as a new type of fluorescent chemosensor for efficient sensing and separation of Zn^2+ ^in ethanol. The inorganic-organic hybrid fluorescent chemosensor DTH-Fe_3_O_4_@SiO_2 _was able to recognize and adsorb Zn^2+ ^with a selective and sensitive fluorescence response in ethanol. The magnetic separation capability of Fe_3_O_4_@SiO_2 _NPs and the reversibility of the combination between DTH-Fe_3_O_4_@SiO_2 _and Zn^2+ ^would also provide a simple route to separate Zn^2+ ^from the environment (Figure 6, inset).

## Abbreviations

APTES: (3-aminopropyl)triethoxysilane; DLS: dynamic light scattering; DRIFT: diffuse-reflectance infrared Fourier transform; DTH: 3,5-di-tert-butyl-2-hydroxybenzaldehyde; DTH-APTES: 2,4-di-tert-butyl-6-((3-(triethoxysilyl)propylimino)methyl)phenol; EDX: energy-dispersive X-ray spectrometer; NPs: nanoparticles; TEM: transmission electron microscopy; TEOS: tetraethyl orthosilicate; TGA: thermal gravimetric analysis; XRD: X-ray power diffraction.

## Competing interests

The authors declare that they have no competing interests.

## Authors' contributions

YW supervised and participated in all the studies and wrote this paper. XP conceived the study and participated in its design. JS participated in the synthesis of the nanoparticles and the testing of fluorescence property. XT, JJ, and WL participated in the revision of the manuscript. All authors read and approved the final manuscript.

## Supplementary Material

Additional file 1 **Characterization and properties of DTH-Fe_3_O_4_@SiO_2_**. Figure S1, XRD patterns of Fe_3_O_4 _core; Figure S2, TGA curves of Fe_3_O_4_@SiO_2 _(a) and DTH-Fe_3_O_4_@SiO_2 _(b); Figure S3, selectivity of DTH-Fe_3_O_4_@SiO_2 _for Zn^2+ ^in the presence of other metal ions in ethanol; and Table S1, the loading of DTH-APTES in the Fe_3_O_4_@SiO_2 _NPs as estimated by different methods. Click here for file
