# Supplementary material for: Highly selective fluorescent chemosensor for Zn2+ derived from inorganic-organic hybrid magnetic core/shell Fe3O4@SiO2 nanoparticles
Source: Nanoscale Res Lett. 2012 Jan 25;7(1):86. doi: 10.1186/1556-276X-7-86 (PMC3299599; doi:10.1186/1556-276X-7-86)
Supplement: Additional file 1 — Characterization and properties of DTH-Fe3O4@SiO2. Figure S1, XRD patterns of Fe3O4 core; Figure S2, TGA curves of Fe3O4@SiO2 (a) and DTH-Fe3O4@SiO2 (b); Figure S3, selectivity of DTH-Fe3O4@SiO2 for Zn2+ in the presence of other metal ions in ethanol; and Table S1, the loading of DTH-APTES in the Fe3O4@SiO2 NPs as estimated by different methods. [file 1556-276X-7-86-S1.DOC]

### Supporting Information for

**Highly selective fluorescent chemosensor for Zn^2+^ derived from inorganic-organic hybrid magnetic core/shell Fe_3_O_4_@SiO_2_ nanoparticles**

### Yujiao Wang, Xiaohong Peng, Jinmin Shi, Xiaoliang Tang, Jie Jiang, Weisheng Liu^§^

Key Laboratory of Nonferrous Metals Chemistry and Resources Utilization of Gansu Province and State Key Laboratory of Applied Organic Chemistry, College of Chemistry and Chemical Engineering, Lanzhou University, Lanzhou 730000, P. R. China

To obtain the mean number of DTH–APTES per nanoparticle, the weight ratio of DTH-APTES, Fe and Si should be measured by TGA and EDX analysis. Besides, we also assumed that the number of magnetic nanoparticles equaled to the Fe_3_O_4_ cores. Hence, the number of DTH–APTES molecules grafted by one particle can be calculated as following.

$∵\frac{m_{Fe}}{m_{Si}}=\frac{2.63\%}{38.94\%}$ (from EDX analysis)

$$\therefore\frac{m_{Fe_{3}O_{4}}}{m_{SiO_{2}}}=\frac{3.63}{83.3}$$

Since the average radio of Fe_3_O_4_ cores was measure to be 10 nm in TEM images, its mass can be obtained:

$$m_{Fe_{3}O_{4}}=\rho_{Fe_{3}O_{4}}\frac{4}{3}\pi R_{_{Fe_{3}O_{4}}}^{3}$$

$$m_{Fe_{3}O_{4}@SiO_{2}}=m_{Fe_{3}O_{4}}+m_{SiO_{2}}$$

$∵\frac{m_{DTH-APTES}}{m_{Fe_{3}O_{4}@SiO_{2}}}=\frac{7.64\%}{100\%-7.64\%}$(from TGA analysis)

$$\therefore N_{DTH-APTES}=\frac{n_{DTH-APTES}}{N_{A}}=\frac{m_{DTH-APTES}/M_{DTH-APTES}}{N_{A}}$$

Whereas *n*, *m* and *M*$w$ means the amount of substance, mass and molecular weight, respectively. The$\rho$, *R* and *N_A_* means the density of Fe_3_O_4_, the radio of Fe_3_O_4_ core and Avogadro's constant.


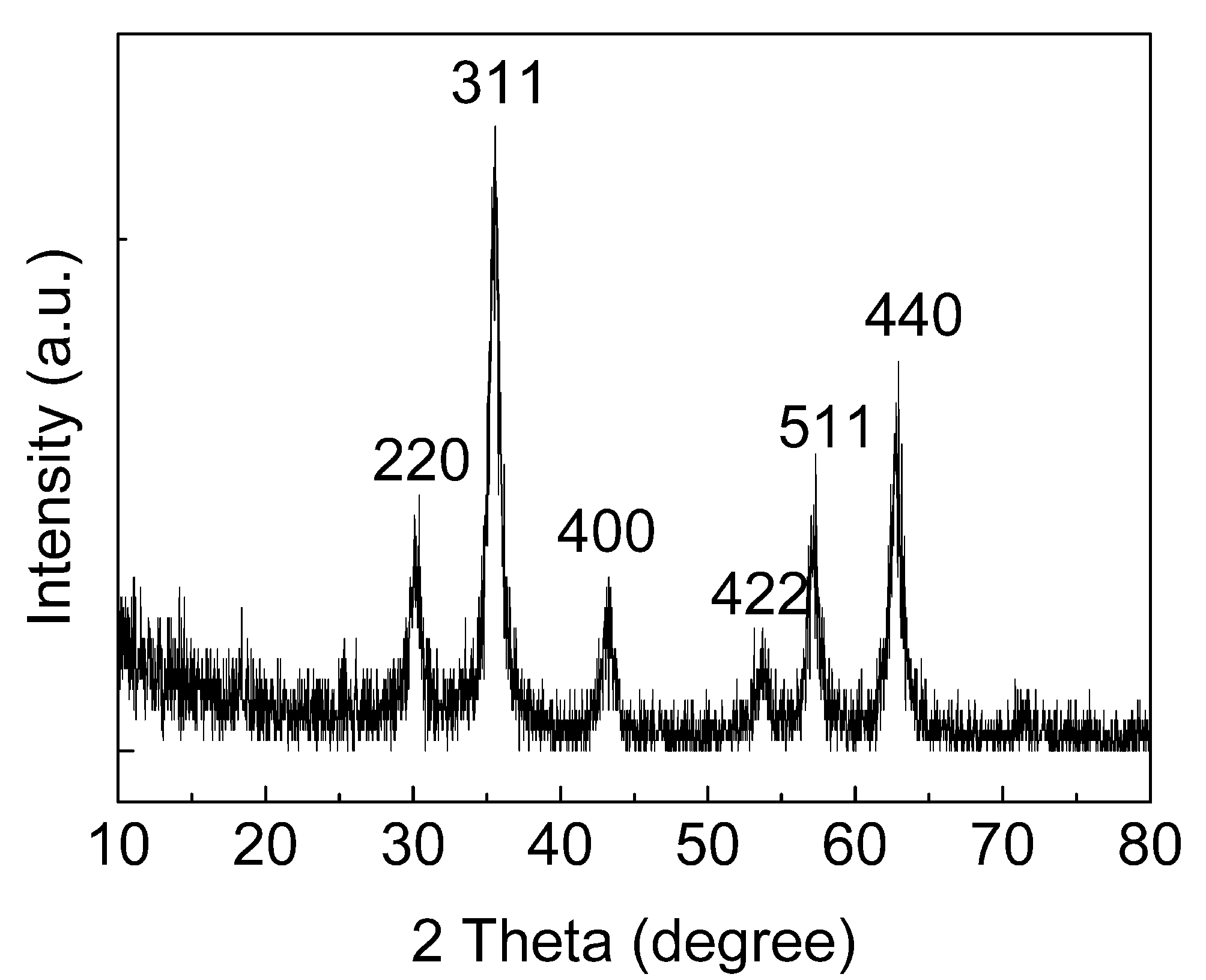


**Figure S1** – **XRD patterns of Fe_3_O_4_ core.**

###
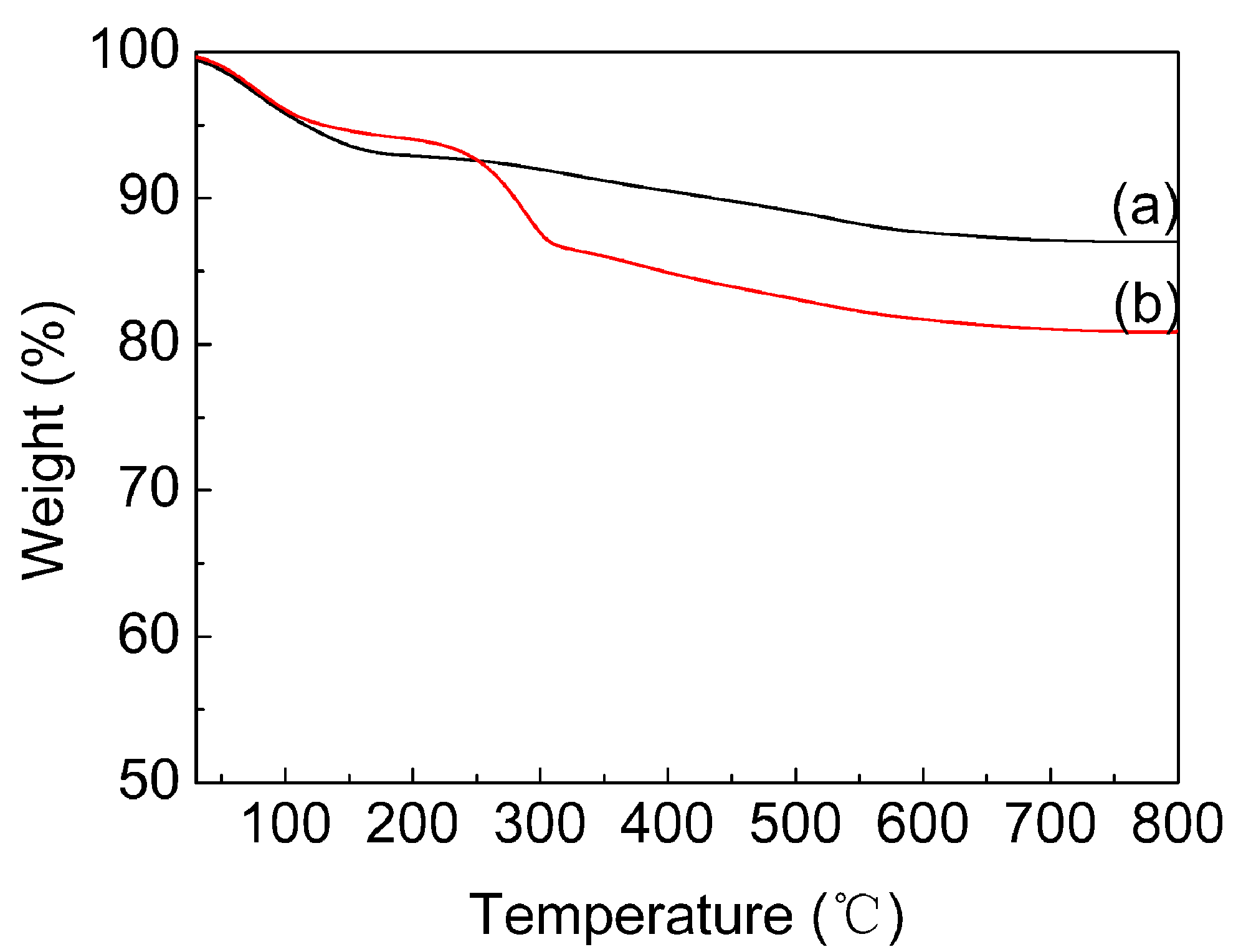


### Figure S2 – TGA curves of Fe_3_O_4_@SiO_2_ (a) and DTH–Fe_3_O_4_@SiO_2_ (b).

The TGA analysis provides further evidence for functionalization of Fe_3_O_4_@SiO_2_ NPs with DTH (Figure S2). TGA results show weight losses of 14.01, 21.65% at 800°C for pristine Fe_3_O_4_@SiO_2_ NPs and DTH–Fe_3_O_4_@SiO_2_ NPs, respectively. The difference in weight loss of Fe_3_O_4_@SiO_2_ NPs and DTH–Fe_3_O_4_@SiO_2_ NPs is attributed to the presence of DTH–APTES molecules on the surface of the Fe_3_O_4_@SiO_2_ NPs.

**Table S1. The loading of DTH-APTES in the Fe_3_O_4_@SiO_2_ NPs as estimated by different methods.**

| Method | Loading of DTH-APTES  [wt%] | Corresponding concentration  [×10^-5^ mol·L^-1^] |
| --- | --- | --- |
| Thermogravimetry  Element analysis | 7.64  7.96 | 5.24  5.45 |

It is shown in Table S1 that 7.96% of DTP-APTES was loaded onto the Fe_3_O_4_@SiO_2_. This value is strongly consistent with the value obtained from thermogravimetric analysis (TGA; Fig. S2).

###
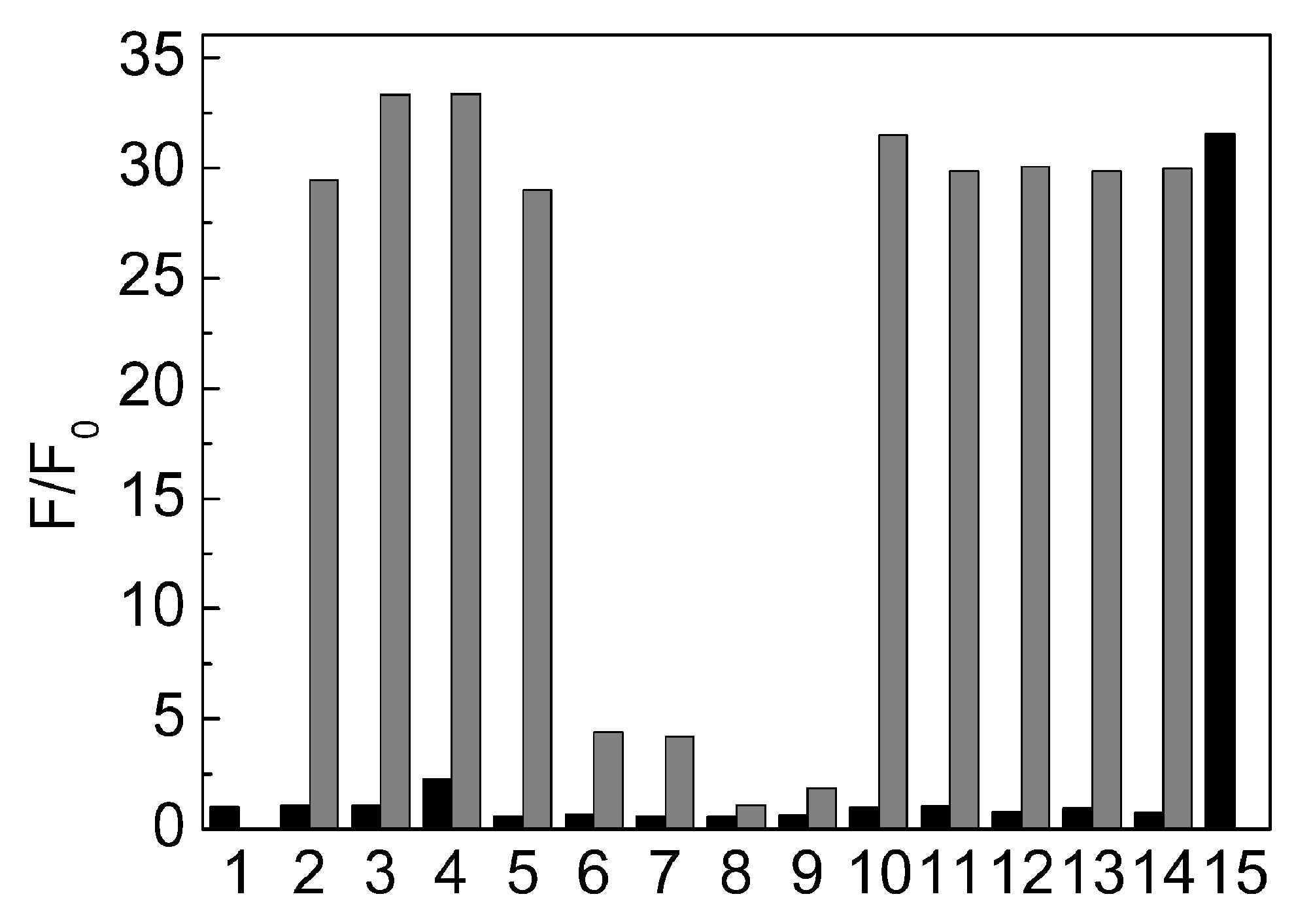


### Figure S3 – Selectivity of DTH–Fe_3_O_4_@SiO_2_ for Zn^2+^ in the presence of other metal ions in ethanol.

λ*ex* = 397 nm. The response is normalized with respect to the background fluorescence of the free ligand (F_0_). Black bars represent the addition of an excess of the appropriate metal ion (100 μM for 1, Blank; 2, Ag^+^; 3, Ca^2+^; 4, Cd^2+^; 5, Co^2+^; 6, Cr^3+^; 7, Cu^2+^; 8, Fe^3+^; 9, Hg^2+^; 10, K^+^; 11, Mg^2+^; 12, Mn^2+^; 13, Na^+^; 14, Ni^2+^; 15, Zn^2+^) to a 0.3 g/L solution of DTH–Fe_3_O_4_@SiO_2_. Gray bars represent the subsequent addition of 100 μM Zn^2+^ to the solution.

The competition experiments indicate that the presence of most metal ions, especially Ca^2+^, K^+^, Mg^2+^, Na^+^, which are abundant in biological environment, have negligible effect on Zn^2+^ sensing. Since Cr^3+^, Cu^2+^, Fe^3+^, Hg^2+^ also appear to bind DTH–Fe_3_O_4_@SiO_2_ sensors, they quench the fluorescence of the Zn^2+^–DTH–Fe_3_O_4_@SiO_2_, owing to an electron or energy transfer between the metal cation and fluorophore known as the fluorescence quenching mechanism.
